# A systematic review on the effectiveness of back protectors for motorcyclists

**DOI:** 10.1186/s13049-016-0307-3

**Published:** 2016-10-04

**Authors:** Rafael Ekmejian, Pooria Sarrami, Justine M. Naylor, Ian A. Harris

**Affiliations:** 1 South Western Sydney Clinical School, University of New South Wales (UNSW), Sydney, Australia; 2 Institute of Trauma and Injury Management, New South Wales Agency for Clinical Innovation, Sydney, Australia; 3 South Western Sydney Local Health District, Liverpool Hospital, Liverpool, Australia

**Keywords:** Protective clothing, Motorcycle, Motorbike, Back protector, Spine protector, Protective armour

## Abstract

**Background:**

Motorcyclists are a vulnerable road-user population who are overrepresented in traffic injuries. Utilisation of back protectors may be an effective preventive measure for spine injuries in motorcyclists. Since use of back protectors is increasing it is important that clinical evidence supports their use. The study aimed to investigate the current evidence on the ability of back protectors to reduce the rate of back injuries and patient mortality in motorcycle crashes.

**Methods:**

A systematic literature search was conducted using various electronic databases. Systematic reviews, randomised controlled trials, controlled clinical trials, cohort studies, case series and case reports were included Opinion pieces and laboratory or biomechanical studies were excluded. Back protectors and spine protectors were included as the intervention; neck braces and speed humps were excluded. The target outcomes were any injuries to the back or death. Only English language studies were included.

**Results:**

The search strategy yielded 185 studies. After excluding 183 papers by title and abstract and full-text evaluation, only two small cross-sectional studies were included. Foam inserts in motorcycle jackets and non-standard clothing may possibly be associated with higher risk of injuries, while hard shell and standard back protectors may possibly be associated with a reduced rate of back and spinal injury.

**Conclusion:**

This systematic review highlighted lack of appropriate evidence on efficacy of back protectors. Based on limited information, we are uncertain about the effects of back protectors on spinal injuries. Further research is required to substantiate the effects of back protectors on mortality and other injuries to the back.

**Electronic supplementary material:**

The online version of this article (doi:10.1186/s13049-016-0307-3) contains supplementary material, which is available to authorized users.

## Background

Due to their relative lack of protection, motorcyclists comprise a vulnerable road-user population who are overrepresented in traffic injuries [1]. Compared to car users, motorcyclists are at increased risk of crashing and are more likely to sustain serious injuries or die in a crash. Indeed, the risk of serious injury and death has been estimated to be as high as 30–35 times greater than car driving [2].

While the rate of spinal cord injuries has decreased in car users, the rate has increased amongst motorcyclists [3] Spinal cord injuries are the most disabling back injuries with a 25.8 % mean disability weight and almost inevitably resulting in lifelong consequences [4–6]. Furthermore, spinal injuries usually affect young people, thus significant economic burden is incurred through loss of their most financially productive years [7, 8].

Utilisation of back protectors may be an effective preventive measure for back injuries including spinal cord injuries in motorcyclists. Back protectors were initially designed for racing sports to reduce abrasion injuries caused by sliding on the road, however, their design evolved for use in normal traffic conditions [9]. They are available as an armour which is either strapped to the body or attached to the insides of motorcycle jackets and can extend from the upper thoracic to lower lumbar spine [10]. The addition of a foam inner liner to the traditional synthetic hard shell was designed to add shock absorption to the abrasion-resistant shell thereby theoretically preventing injuries by reducing forces transmitted in direct blows to the back and spine [9, 11]. Indeed, the European standard EN1621-2, which is the sole compulsory standard to which European back protectors must adhere, assesses ergonomics and impact force reduction rather than abrasion resistance [12, 13].

Through their shock absorptive and abrasion resistant qualities, back protectors are intended to protect the tissues of the back, shoulder blades, ribs and spine [11]. Some designs add sacral portions or kidney protectors to extend protection to this region [10]. Furthermore, some back protectors limit spinal extension thereby theoretically preventing spinal injury through hyperextensive forces [10].

Due to the increased availability of body armour in the market there has been increased usage among motorcyclists. Surveys comparing the use of back protectors among motorcyclists in Australia reflect this upward trend; while only 19 % of motorcyclists used back protectors in 2001, this increased to 44 % use for commutes and 47 % for recreational motorcycling in a 2006 follow-up survey [14]. Despite their increased use, the theoretical evidence for the use of back protectors is contentious. Indeed, it is thought that they cannot protect against the most serious back and spinal injuries which occur due to twisting and bending to the back [14]. Furthermore, the use of back protectors may be associated with rider discomfort and fatigue thus impairing the motorcyclist’s ability to ride safely. It is therefore important to determine whether any theoretical protection afforded by back protectors translates into benefit.

This study aimed to review the evidence in support of back protectors for the prevention of back injuries in motorcyclists. Here, back injuries are defined as musculoskeletal injuries including injuries to thoracic and lumbar spine. We also aimed to investigate whether back protectors reduce mortality in motorcycle crashes.

## Methods

In order to answer the research questions of this study a systematic literature search was conducted using the electronic databases MEDLINE, EMBASE, CINAHL, Cochrane Central Register of Controlled Trials, and Google Scholar. A hand search of the reference list of the included articles and relevant books and book chapters was also undertaken. The PRISMA statement guided the approach [15] (Additional file 1).

To be considered for inclusion into this systematic review, eligible studies were those that sampled a population of adults who were either motorcycle riders or pillion passengers. Systematic reviews, randomised controlled trials, controlled clinical trials, cohort studies, case series and case reports were included. Opinion pieces and laboratory or biomechanical studies were excluded. Back or spine protectors were included as the preventative intervention. While neck braces attempt to provide protection to the spine in motorcycle crashes, they were excluded as they operate via a different mechanism and their efficacy is not the focus of this particular study. Furthermore, studies were excluded if they evaluated ‘speed humps’ as a back protective mechanism. These are areas of convex padding incorporated into the posterior of leather jackets and aim to increase aerodynamics rather than provide any protection [10]. The target conditions being examined by this study included back injuries (musculoskeletal injuries including injuries to thoracic and lumbar spine), while injuries to other regions were excluded. There was no time limitations. Lastly, only studies available in English were included. The process of screening, inclusion and exclusion based on title and abstract or full text, was undertaken by two independent researchers (RE and PS). Discrepancies between the two authors were managed via discussion between the two authors.

## Results

### Study selection

**Fig. 1:**
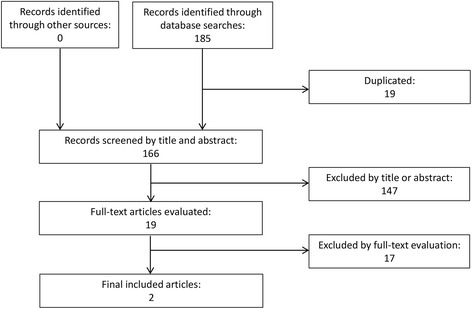
Flow diagram of study selection

While the search strategy initially yielded 185 studies, 19 of these were duplicates. Of the remaining 166 studies, 147 were subsequently excluded by title and abstract, due to non-relevancy of topic or deficiency in methods. The remaining 19 studies were assessed by full-texts and only two studies were found studying the effect of back protectors on back injuries. No studies were found to assess the effect of back protectors on mortality. Seventeen papers studies were evaluated by full text and were excluded because they did not specifically assess back protectors for motorcyclists. The study selection process is summarised in Fig. 1 and inclusion and exclusion criteria and search strategy are presented in the (Additional file 2).

### Study design

The two included studies were both cross-sectional analyses conducted by de Rome et al. [16] in Australia and Giustini et al. [17] in Italy. The Australian study conducted by de Rome et al. [16] did not solely investigate the efficacy of back protectors but was aimed at quantifying the association between several types of protective clothing and injury in crashes. Regarding the use of back protectors, the study sought to determine whether foam inserts in motorcycle jackets or separate back armour affected the risk of back or spine injuries (all soft tissue injuries, open wound injuries, fractures and any injuries) compared to no back protection. The study characteristics are summarised in Table 1. Of the 212 riders or passengers involved in motorcycle crashes causing injury or vehicle damage, 126 (59.4 %) were identified through hospital records, 75 (35.4 %) from local crash repairers and 9 (4.2 %) were self-referred [16].

**Table 1:** Characteristics of the included studies

	De Rome et al. 2011 [16]	Giustini et al. 2014 [17]
Study design	Cross-sectional	Cross-sectional
Study location and time	Australia, 2008	Italy, 2011–2013
Sample size	212	2,319
Participant identification	- Hospital records (60 %) - Local crash repairers (36 %) - Self-referred (4 %)	Collaboration of the Italian National Institute of health with the National Traffic Police
Data collection	Baseline	Baseline + 30d after hospitalisation
Intervention	1. Motorcycle jacket 2. Motorcycle gloves 3. Motorcycle pants 4. Motorcycle boots 5. Helmet 6. Motorcycle back protector (Foam insert in jacket & back armour)	1. Hard-shell back protectors 2. Jacket or vest with an air bag 3. Uncertified Protective clothing;
Outcome	Back/spine injuries	Spine fracture and spinal cord injury

The only significant finding reported by the study was that foam inserts increased the adjusted relative risk of any injury (RR 2.16, *p* ≤ 0.05) [16]. In contrast, separate back armour had a reduced unadjusted and adjusted relative risk of any injuries and all soft tissue injuries, however, these findings were non-significant [16]. The findings of this study are summarised in Table 2. As also declared by the authors, the lack of effect of back protectors may reflect the insufficient sample size of their study. Indeed, data on the effect of back protectors to the adjusted relative risk of open wounds and fractures was not presented due to small numbers and convergence issues [16].

**Table 2:** Summary of findings of de Rome et al. [16] on the effects of back protection adjusted relative risk of back injuries

Motorcycle back protector	Number (Total *n* = 212)	Any injury (%)	Adjusted relative risk
No	75	10.7 %	Reference
Foam insert in the back of jackets	97	21.6 %	2.16 (*p* ≤ 0.05)
Back armour	40	7.5 %	0.77 (Not Significant)

The Italian study by Giustini et al. [17] sought to assess the effectiveness of back protectors in reducing the number and severity of spinal injuries. The 2319 riders or passengers involved in motorcycle crashes were recruited via a register of traffic police interventions.

In contrast to de Rome et al. [16], the Giustini et al. study contained a much larger sample size and they found statistically different rates in spinal injury based on the level of protection (Table 3). In this study, hard-shell back protectors included only those compliant with the EN1621-2/12 standard while protective clothing designates those that have not reached the standards of this certification [17]. However, since this study analysed hard-shell back protectors and jackets/vests with safety airbags together, it cannot be determined whether a hard-shell protector is more effective at reducing spinal injuries than airbags. These airbags are incorporated in motorcycle jackets and are deployed when a rider falls off their motorcycle. Nevertheless, there was an increased Mantel-Haenszel odds ratio for spinal cord fracture and injury when uncertified or no protection was compared to certified hard-shell back protectors or airbags (OR = 2.72, *P* = 0.049, 95 % CI: 1.00–7.74) [17].

**Table 3:** Summary of findings of Giustini et al. [17] on the effect of back protection to risk of spinal injury

	No back protection	Protective clothing	Hard-shell or airbag
Spinal injury	52 (59 %)	24 (27 %)	12 (14 %)
No spinal injury	293 (67 %)	67 (15 %)	80 (18 %)

Giustini et al. [17] incorporated analysis of motorcycle and moped crashes together. This was despite noted differences in mechanisms of injury between the two groups with moped crashes more likely to be involving other vehicles and less likely to be due to a loss of control. This reduces the generalisability of the study as a measure of the effectiveness for back protectors for motorcycle accidents only.

Although De Rome et al. [16] sampled patients from local crash repairers and self-referred cases as well, the majority of their sample (60 %) was obtained from hospital records which is likely to bias the sample towards more severe crashes and injuries. This may involve more cases where back protectors had failed to work while excluding those where back protectors proved effective.

Furthermore, selecting for motorcycles undergoing crash repairs may bias the sample towards higher impact motorcycle crashes and therefore more severe injuries. Indeed, studies of car and motorcycle crashes have shown a correlation between crash severity (defined by damage to property) and injury severity in the acute phase [16, 17]. In addition, lower impact crashes may be less likely to cause damage to a motorcycle, however, most motorcycle crashes don’t involve high speed [18]. Hence, it is possible that obtaining participants through crash repairers is excluding those involved in slower speed crashes. This is pertinent as protective clothing is perceived to be more effective for reducing injuries for low impact crashes [19].

The involvement of repair services in recruitment, where they received a recruitment fee to obtain consent and contact details from customers involved in crashes, also increases the susceptibility to selection bias. These non-research affiliated repairers may have been less stringent with their recruitment methods.

De Rome et al. [16] list the combination of both injury and non-injury motorcycle crashes as a strength of the study, as previous studies utilising injury and/or police reported crashes were biased towards more severe crashes. However, combining injury and non-injury crashes does not necessarily achieve a proportion of injury to non-injury that accurately represents road motorcycle crashes. Indeed, the authors themselves note that the number of injury crashes in the sample is greater and non-injury crashes substantially less than those recorded by police. The study is therefore likely to underestimate any benefits of back protectors.

Baseline data were collected from face to face interviews of patients 2 weeks following their crash. However, for subjects retrieved through crash repairers, a limitation is that there was no corroboration of their clinical history. Given the absence of independent investigation to corroborate the participants’ reports, there is an increased susceptibility to response bias.

The Giustini et al. review is also susceptible to sampling bias as analysis as spinal injuries were determined in hospitalised or deceased victims who had a diagnosis of spinal injuries according to the large groups of ICD-9-CM diagnoses (diagnosis codes: 805, 806, 839) [17]. Hence, the study only analyses the effectiveness of back protectors in reducing the most severe spinal cord injuries. Subjects were also identified by a register of traffic police records, hence, sampling for more severe crashes which required police attendance, and underreporting of crashes could have affected their sample.

## Discussion

Considering the differences between these studies it was not possible to pool their data for meta-analysis. The lack of studies assessing back protectors and the lack of significant data presented by de Rome et al. [16] reflect weak evidence supporting the use of back protectors to reduce back injuries in motorcycle crashes. While de Rome et al. [16] found that foam inserts significantly increased the adjusted relative risk of any injury (RR 2.16, *p* ≤ 0.05), this finding is unlikely to have clinical applicability given that it pools less serious injuries such as superficial soft tissue injuries with potentially more serious injuries such as fractures. Clinical relevance is further diminished by susceptibility to selection and response bias in addition to the limitations of a cross-sectional analysis. While Giustini et al. [17] demonstrate that certified safety protection may reduce risk of serious spinal cord injury, these finding are limited in that they do not distinguish between motorcycle and moped crashes, which can vary in their speeds, or in the protection used such as airbags and hard shell back protection. Furthermore, this study did not assess any injuries to the back apart from spinal injuries.

For both studies, it would have been useful to adjust for the mechanism of back injury. This would have allowed a determination of whether back protectors are more effective in particular crash circumstances. In a study of 696 crashes of motorized two-wheelers, it was found that soft tissue injury risk to the back mainly related to direct impact to the road resulting in shearing and gliding. Furthermore, fractures to the spine mostly occurred in isolated falls combined with sliding of the body on the ground under compression and bending load [9]. Hence, the benefits of an abrasion resistant hard shell are unlikely to be seen unless the motorcyclist’s back is forced along a road surface in a crash. Furthermore, while back protectors are unable to protect against most spinal injuries, which are caused by bending and torsional forces, it would be useful to see whether back protectors fulfil their designed function of reducing injuries by protecting against direct blows to the back [20]. Furthermore, neither study incorporates analysis of kinematic energy at the time of accident. It is necessary to explore if back protectors lose efficacy in higher speed crashes.

It may also have been beneficial to distinguish between risks of back injuries based on severity. While the prevention of serious injuries is likely the most important factor in the purchase of a back protector, the ability to prevent more minor injuries might still warrant its use. Given that protection against spinal injuries causing neurological compromise is likely a key consideration in the purchase of a back protector, it would have been useful to determine whether back protectors have an impact on preventing neurological compromise.

A strength of the study by de Rome et al. [16] was that it differentiated between jacket inserts and separate back protectors. Jacket inserts often have a reduced surface area and protect a smaller area of the back. Furthermore, in order to minimize size jacket inserts often utilise thinner foam which theoretically provides less shock absorptive capacity. In addition, the abrasion resistant synthetic hard shell is often foregone thereby theoretically reducing abrasion resistance. Indeed differences in protection may be suggested by the differences in adjusted relative risk seen between the two designs.

Studies on protective clothing have shown significant discrepancies in quality across different manufacturers [11]. In order to minimise the influence of such manufacturing discrepancies on the level of protection afforded, a significant advantage of Giustini et al. [17] was that the hard-shell back protectors and airbags category only included devices which were adherent to European standards EN1621-2. For a hard-shell back protector to adhere to this standard, average peak forces transmitted through the back protectors must be reduced to below 9kN during mechanical testing [20]. By only including back protectors adherent to these standards researchers would be able to limit differences in protection due to design inadequacies. It would additionally be beneficial to research back protectors with and without synthetic hard shells to determine if this design feature has any benefit to abrasion resistance.

Finally, it is notable that the rate of injury was increased in motorcyclists who used jackets with foam inserts [16] or those with non-standard protective clothing [17] in comparison with motorcyclists with no back protection. Here speed might be a confounding factor. Those with non-standard protective clothing might be involved in crashes with higher speeds in comparison with those with no protection. Therefore, as Giustini et al. have suggested it is important to explore the kinetic energy (speed of the vehicles) at the time of crashes [17]. It will be also interesting to see if motorcyclist could have an overestimation of the protection that protective gears can present. If such a false sense of protection exists, it can lead to an increase of risk-taking that can overweigh the protection of gears.

## Conclusions

This systematic review indicates a lack of evidence concerning the efficacy of back protectors in the prevention of injury in motorcyclists. Based on limited information, we are uncertain about the effects of back protectors on spinal injuries. Further research is required to substantiate the effects of back protectors on mortality and other injuries to the back and to investigate the efficacy of various types of back protectors. Subsequent analysis should investigate the role of injury mechanism to determine whether back protectors are more useful under particular crash circumstances. Lastly, future studies should investigate the role of back protectors on the severity of injury outcomes to the back.

## Additional files

Additional file 1: PRISMA 2009 Checklist. (DOC 63 kb)
Additional file 2: Search strategy. (DOC 34 kb)
