# Supplementary material for: A systematic review on the effectiveness of back protectors for motorcyclists
Source: Scand J Trauma Resusc Emerg Med. 2016 Oct 4;24:115. doi: 10.1186/s13049-016-0307-3 (PMC5050611; doi:10.1186/s13049-016-0307-3)
Supplement: Additional file 2: — Search strategy. (DOC 34 kb) [file 13049_2016_307_MOESM2_ESM.doc]

**Search strategy:**

**Inclusion and exclusion criteria u**tilised in this systematic review

| Inclusion Criteria | | Exclusion Criteria |
| --- | --- | --- |
| **Sampling** | Adults  Motorcycle Riders  Motorcycle Pillion Passengers | Children |
| **Study type** | Systematic Reviews  Randomised Controlled Trials  Cohort Studies  Case-control Studies  Cross-sectional Surveys  Case Studies | Opinions  Discussions  Letters |
| **Protective equipment** | Back Protectors  Spine Protectors | Speed Humps  Neck Braces |
| **Target condition** | Any Injuries to the Back | Injuries Affecting Body Regions Apart from the Back |
| **Language** | English | Non-English Languages |

**Search strategy utilised for each database in this study**

| **Database** | **Search strategy** |
| --- | --- |
| **Medline** | 1. Protective Clothing/ and Motorcycles/ (both as subject headings)  2. Limit 1 to English |
| **Embase** | 1. motorcycle/ and protective clothing/ (both as subject headings)  2. Limit 1 to English |
| **Cochrane** | MeSH descriptor: [Motorcycles] |
| **Google scholar** | 1. ("back protector" OR "spine protector" OR "protective armour" OR "protective armor") AND (motorcycle OR motorbike)  2. filter by articles (patents not included) |
| **Cinahl** | 1. ("back protector" OR "spine protector" OR "protective armor" OR "protective armour") AND (motorcycle OR motorbike)  2. Limit 1 to English |
